# Imagery in the aftermath of viewing a traumatic film: Using cognitive tasks to modulate the development of involuntary memory

**DOI:** 10.1016/j.jbtep.2011.10.008

**Published:** 2012-06

**Authors:** Catherine Deeprose, Shuqi Zhang, Hannah DeJong, Tim Dalgleish, Emily A. Holmes

**Affiliations:** a Department of Psychiatry, Warneford Hospital, University of Oxford, Oxford OX3 7JX, UK; b MRC Cognition and Brain Sciences Unit, 15 Chaucer Road, Cambridge CB2 7EF, UK

**Keywords:** Involuntary memory, Intrusions, Memory consolidation, Mental imagery, Visuospatial working memory, Episodic memory, Autobiographical memory

## Abstract

**Background and objectives:**

Involuntary autobiographical memories that spring unbidden into conscious awareness form part of everyday experience. In psychopathology, involuntary memories can be associated with significant distress. However, the cognitive mechanisms associated with the development of involuntary memories require further investigation and understanding. Since involuntary autobiographical memories are image-based, we tested predictions that visuospatial (but not other) established cognitive tasks could disrupt their consolidation when completed post-encoding.

**Methods:**

In Experiment 1, participants watched a stressful film then immediately completed a visuospatial task (complex pattern tapping), a control-task (verbal task) or no-task. Involuntary memories of the film were recorded for 1-week. In Experiment 2, the cognitive tasks were administered 30-min post-film.

**Results:**

Compared to both control and no-task conditions, completing a visuospatial task post-film reduced the frequency of later involuntary memories (Expts 1 and 2) but did not affect voluntary memory performance on a recognition task (Expt 2).

**Limitations:**

Voluntary memory was assessed using a verbal recognition task and a broader range of memory tasks could be used. The relative difficulty of the cognitive tasks used was not directly established.

**Conclusions:**

An established visuospatial task after encoding of a stressful experience selectively interferes with sensory-perceptual information processing and may therefore prevent the development of involuntary autobiographical memories.

## Highlights

► Involuntary memories present a transdiagnostic treatment target. ► Completing cognitive tasks after a stressful event may reduce involuntary memories. ► The visuospatial nature of the cognitive task may be critical in modulating effects.

## 1 Introduction

Cognitive models of autobiographical memory (e.g. Conway & Pleydell-Pearce, 2000) make an important distinction between voluntary and involuntary memory. A voluntary memory, for example, could include deliberately recalling a previous event. An involuntary memory would be a seemingly spontaneous recollection without deliberate intention to bring that event to mind (Anderson & Levy, 2009; Berntsen & Jacobsen, 2008; Johannessen & Berntsen, 2010; Mace, 2007; Mandler, 1994; Richardson-Klavehn, Gardner, & Java, 1994; Schacter, 1987). Involuntary memories are a common phenomenon in healthy adults (Bernsten, 1996; Kvavilashvili & Mandler, 2004). Indeed, Rubin and Berntsen (2009) report that frequencies of voluntary and involuntary recollections of significant events are comparable, making the relative lack of research in the area even more remarkable. Involuntary memories have broad relevance to experimental psychopathology and are highlighted as a critical transdiagnostic treatment target across a range of disorders (Brewin, Gregory, Lipton, & Burgess, 2010; Holmes & Hackmann, 2004; Holmes & Mathews, 2010).

Involuntary memories are typically sensory-perceptual rather than verbal (Arntz, de Groot, & Kindt, 2005; Brewer, 1996; Conway, 1990, 2005; Conway, Meares, & Standart, 2004; Conway & Pleydell-Pearce, 2000), relate to specific events rather than summaries across several events (Schlagman & Kvavilashvili, 2008) and are more frequently negative than positive (Bywaters, Andrade, & Turpin, 2004; Walker, Skowronski, Gibbons, Vogl, & Ritchie, 2009). However, laboratory research in experimental psychology has predominately focussed on memories associated with deliberate, intentional recollection. The basic cognitive processes underlying the development of involuntary memories are relatively under-explored. Improving our understanding of these processes would advance theoretical frameworks of involuntary memory development and inform evidence-based treatment innovation.

The stressful film paradigm is used to induce involuntary memories in healthy volunteers as an analogue of real-life experience and subsequent memory formation (Horowitz, 1969). Participants are shown a short aversive film in controlled laboratory conditions, allowing testing of specific hypotheses relating to subsequent involuntary memories (see Holmes & Bourne, 2008 for a review).

Experiments using the stressful film paradigm show that involuntary memories may be vulnerable to interference at encoding using specific cognitive tasks. Completing visuospatial working memory tasks *during* film viewing reduces subsequent involuntary memories (Holmes, Brewin, & Hennessy, 2004; Stuart, Holmes, & Brewin, 2006). Conversely, performing other tasks during the film, such as counting backwards, has been shown to increase involuntary memories relative to no-task controls in some studies (Bourne, Frasquilho, Roth, & Holmes, 2010; Holmes et al., 2004) but not others (e.g., Krans, Naring, Holmes, & Becker, 2009). Broadly, these findings support working memory models predicting that modality-specific, limited capacity resources are required for the encoding of involuntary memories (Andrade, Kemps, Werniers, May, & Szmalec, 2002; Baddeley & Andrade, 2000; Kavanagh, Freese, Andrade, & May, 2001; Kemps & Tiggemann, 2007; Krans, Naring, Holmes, & Becker, 2010; May, Andrade, Pannaboke, & Kavanagh, 2010). Here, we examine for the first time to our knowledge, whether established working memory tasks from the cognitive science literature can interfere with the development of involuntary memories when performed post-encoding of a stressful event. The current studies expand on previous work investigating the role of concurrent experimental task manipulations during the encoding stage on the development of involuntary memories (Bourne et al., 2010; Holmes et al., 2004; Stuart et al., 2006) by exploring the impact of completing task manipulations in the memory consolidation phase (following film viewing).

Memory consolidation refers to the process of stabilization following initial acquisition of information, during which memories are subject to interference for a period of 6-h (Nader, 2003; Walker, Brakefield, Hobson, & Stickgold, 2003). Thus, we previously predicted that completing a visuospatial task after viewing a stressful film would interrupt the consolidation of the sensory-perceptual information required for the development of involuntary memories via competition for the same limited cognitive resources (Holmes, James, Coode-Bate, & Deeprose, 2009; Holmes, James, Kilford, & Deeprose, 2010). In accordance with this, we found that playing the visuospatial computer game “Tetris” (Green & Bavelier, 2003; Sims & Mayer, 2002; Stickgold, Malia, Maguire, Roddenbury, & O’Connor, 2000) after a stressful film reduced involuntary memories relative to a no-task control condition (Holmes et al., 2009) and relative to a both a no-task control condition and a comparable verbal computer game “Pub Quiz” (Holmes et al., 2010).

The “visuospatial hypothesis” predicts that “Tetris” competes for the same sensory-perceptual resources as involuntary memories. We argue that the beneficial effects of “Tetris” in reducing involuntary memory development are attributable to the visuospatial nature of the game rather than providing distraction or enjoyment (Holmes et al., 2010). However, according to a general attention and working memory approach, it is possible that any task could interfere with the development of negative involuntary memories due to loading on the central executive (Engelhard, van den Hout, Janssen, & van der Beek, 2010; Engelhard, van den Hout, & Smeets, 2011; Gunter & Bodner, 2008; van den Hout et al., 2011; van den Hout et al., 2010; van den Hout, Muris, Salemink, & Kindt, 2001; Krans, Naring, & Becker, 2009). Our “visuospatial hypothesis” needs to be tested by examining whether an established visuospatial working memory task (e.g. complex pattern tapping; Baddeley & Andrade, 2000) also serves to reduce the development of involuntary memories in comparison to an established control task (e.g. counting backwords; Vallar & Baddeley, 1982).

We report two studies designed to extend our initial findings (Holmes et al., 2009; Holmes et al., 2010) utilizing working memory tasks previously used in the cognitive psychology literature rather than computer games. In Experiment 1, participants watched the stressful film then immediately completed a visuospatial task (complex pattern tapping), a control-task (backwards counting) or no-task. In Experiment 2, we extended the time-frame from immediately post-film to 30-min post-film.

## 2 Experiment 1

The visuospatial task involved tapping a five-key pattern on a keyboard concealed from view (Moar, 1978) as reported in Holmes et al. (2004) and Morris (1987). The control-task involved counting backwards aloud from specified three-digit numbers (Holmes et al., 2004, Expt. 3; Tree, Longmore, Majerus, & Evans, 2011; Vallar & Baddeley, 1982). The main outcome was the number of involuntary memories of the film over 1-week. We predicted participants in the visuospatial condition would have fewer involuntary memories relative to both the control-task and no-task task conditions.

### 2.1 Method

#### 2.1.1 Overview and procedure

All participants completed baseline assessments, pre-film mood ratings and received standardized training on both the visuospatial task and control-task before watching a stressful film. During the film, participants were asked to sit still and pay close attention, imagining that they were “a bystander” present and involved at the scenes of the events being shown. They were asked not to look away or shut their eyes as they would be asked questions about the contents of the film later. Immediately after the film, participants completed ratings for post-film mood, attention paid to film and personal relevance of the film and then completed the assigned experimental task for 10-min. They were then shown how to complete the involuntary memory diary and after 7 days, returned for a follow-up session.

#### 2.1.2 Participants

Sixty volunteers (39 females), with an age range from 18 to 58 (mean = 27.4) were paid a small fee for participation. Participants were recruited locally via online advertisements. For ethical considerations, recruitment materials mentioned that the film would contain graphic and potentially disturbing images. As part of informed consent, all participants confirmed that they had not received any treatment for a mental health problem, nor were planning to undertake a university examination in the following week. A minimization scheme (Scott, McPherson, Ramsay, & Campbell, 2002; Treasure & MacRae, 1998) was used to allocate participants to experimental groups and to ensure equivalence in age, BDI, and STAI-T.

#### 2.1.3 Stressful film

A 9-min stressful film (based on Holmes et al., 2009) comprised 13 extracts of film footage already in the public domain such as Public Information Films. Five scenes depicted motor vehicle accidents, two scenes depicted surgery, two scenes depicted drowning and four additional scenes included: an electricity pylon accident, a firework explosion, a house fire and bullying. The film was displayed on a 1.4 m × 0.8 m screen approximately 2 m from the viewer via a data projector connected to a computer.

#### 2.1.4 Experimental tasks after the stressful film

Visuospatial tapping task: Participants tapped pre-designated spatial patterns on a keypad with a 5 × 5 array of buttons with their dominant hand (Moar, 1978). Prior to the film, participants received brief training in which they were asked to hold each of the three patterns in their mind’s eye to complete the tapping task as the keypad would be later concealed from view. During this standardized training, the experimenter provided feedback to ensure accurate performance. Immediately post-film, participants received a brief reminder of the task instructions and were informed that the computer would record their responses to assess accuracy. They then began continuous pattern tapping for 10-min without experimenter feedback: Sequence 1 (3-min), Sequence 2 (3-min) and Sequence 3 (4-min). The duration of tapping for each sequence was timed by the experimenter who prompted when to start and finish each one. Responses were recorded via computer and later scored for accuracy.

Control-task: Participants counted backwards aloud in threes from three pre-designated seed numbers (Holmes et al., 2004, Expt. 3; Vallar & Baddeley, 1982). Prior to the film participants received brief training to count aloud backwards. The standardized training involved experimenter feedback. Immediately post-film, participants received a brief reminder of the task instructions and were informed their responses would be audio-taped to assess accuracy. They then began continuous counting for 10-min without experimenter feedback from the following seed numbers: 958 (3-min), 845 (3-min), 969 (4-min). The duration of counting from each seed number was timed by the experimenter who prompted when to start and finish each one. Responses were audio-taped and later scored for accuracy.

No-task: Immediately after the stressful film, participants received standardized instructions that they were to have a short break seated in the laboratory for 10-min, during which time they could think about anything without restriction but not talk with the experimenter.

#### 2.1.5 Measures

At baseline, current depressive symptoms were assessed using the Beck Depression Inventory-II (BDI-II; Beck, Steer, & Brown, 1996) and trait anxiety was assessed using the Spielberger Trait Anxiety Inventory (STAI-T; Spielberger, Gorsuch, Lushene, Vagg, & Jacobs, 1983).

Before and after the film, mood (sadness, hopelessness and depression) was rated by participants using visual analogue scales (Holmes et al., 2009). A composite mood score was calculated for each time-point. After watching the film, participants also rated the personal relevance of the film and how much attention they had paid to the film using similar scales.

Involuntary memories of the film were recorded by participants over 7-days a using paper diary (cf. Holmes et al., 2004; Stuart et al., 2006). Involuntary memories were defined as “mental images from the film” which could occur in any sensory modality (e.g. visual, auditory and so on). Participants were informed that if they deliberately brought the memory to mind or thought about the film verbally, this did not count. Participants were asked to fill in the diary as soon as possible after each involuntary memory and to detail the content (e.g. “seeing someone drowning in the sea”). This allowed the experimenter to verify that each involuntary memory reported related to the film. The total number of involuntary memories was calculated for each participant. This included involuntary memories from all sensory modalities reported by participants although note that as the film was a visual stimulus with sound, subsequent involuntary memories were also in these modalities (rather than taste or smell, for example). The extent to which each participant felt they had been accurate in maintaining their diary was also rated on a visual analogue scale.

### 2.2 Results and discussion

The data were examined for potential univariate outliers. No scores were more than 3 standard deviations from the mean (Tabachnick & Fidell, 1996). An alpha level of 0.05 was used for all statistical tests.

#### 2.2.1 Group allocation, manipulation and compliance checks

There were no significant differences between the groups in age, BDI-II, STAI-T (*F*s < 1) or gender [*χ*^2^ (2, *N* = 60) = 1.76, *p* = .42] (Table 1). Ratings for attention paid to the film or personal relevance of the film did not differ between groups (*F*s < 1; Table 1). However, as predicted, viewing the film resulted in increases in negative mood which were comparable between groups (Table 1). Repeated measures ANOVA on mood confirmed a significant main effect of time, *F*(1, 57) = 32.96, *MSE* = 41.15, *p* < .001, but no main effect of group, *F*(2, 57) = 0.33, *p* = .72, or time × group interaction, *F*(2, 57) = 0.50, *p* = .61.

In terms of experimental task compliance, participants in the visuospatial condition tapped rapidly and accurately. The mean total number of key presses during the 10-min experimental phase was 62 per min (*SD* = 13.8) and an accurate five-key sequence was tapped on 82.2% of occasions (see Table 1). The control-task also demonstrated high levels of performance. The mean number of responses (numbers counted) was 235 (*SD* = 63.7) and the number of errors was 7.7 (*SD* = 9.6) (Table 1) meaning that accurate responses were provided on 96.7% of occasions. For each task, the level of performance in this experiment was comparable to previous studies (Holmes et al., 2004).

There were no differences between groups in the accuracy reported by participants in maintaining the involuntary memory diary over the 7 days [*F*(2, 57) = 1.68, *p* = .20] (Table 1).

#### 2.2.2 Effects of experimental condition

**Fig. 1:**
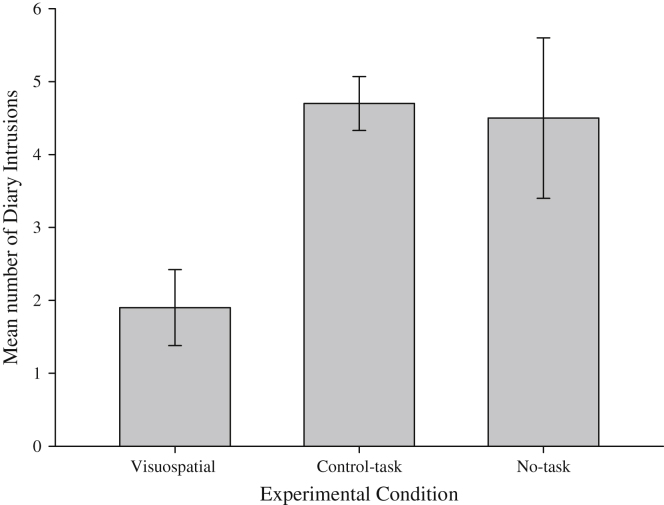
Experiment 1: Number of Involuntary Memories for each Experimental Condition. Error bars represent standard error of the mean.

One way ANOVA confirmed a significant difference in the number of involuntary memories of the stressful film between experimental conditions, *F*(2, 57) = 4.76, *MSE* = 48.8, *p* = .012, $\eta_{p}^{2}$ = 0.14 (Fig. 1). Independent samples *t*-tests indicated significantly fewer involuntary memories in the visuospatial group compared to both no-task [*t*(38) = 2.20, *p* = .034, *d* = 0.66] and to the control-task [*t*(38) = 4.40, *p* ≤ .001, *d* = 1.1]. The no-task and control-task conditions did not significantly differ [*t*(38) = 0.18, *p* = .86].

Thus in summary, Experiment 1 confirmed that participants completing the visuospatial tapping task condition experienced fewer involuntary memories compared to both the control-task and no-task conditions when completed immediately post-film.

## 3 Experiment 2

Experiment 2 aimed to extend the key finding that completing a visuospatial task after watching a stressful film decreases involuntary memories relative to both a no-task and control-task condition, by using a longer time interval of 30-min between film and task. This was predicted to be within the time-window for memory consolidation, given that certain types of memories are initially labile for 6-hrs (Nader, 2003; Walker et al., 2003).

It could be argued that the visuospatial task had reduced involuntary memories not via modality-specific interference but by greater cognitive load. The control-task in Expt 2 was therefore adapted to be more challenging by requiring participants to count out-loud backwards in sevens (Wegner, 1994) rather than threes as in Expt 1. A recognition memory task was included to index voluntary retrieval for the stressful film. The film was extended in length to incorporate a wider range of stressful themes including self-harm and genocide and to develop footage for later fMRI studies (Bourne, Mackay, & Holmes, submitted for publication).

In line with Experiment 1, we predicted that the number of involuntary memories over 1-week after the visuospatial tapping task would be fewer relative to both the no-task and control-task.

### 3.1 Method

#### 3.1.1 Overview and procedure

The procedure closely replicated that used in Experiment 1, with the critical modification being that participants completed the experimental tasks 30-min following the stressful film rather than immediately after the film.

#### 3.1.2 Participants

Seventy-five volunteers (37 females), with an age range from 18 to 59 (mean = 25.5), were paid a small fee for their participation.

#### 3.1.3 Stressful film

The 22-min film contained 14 scenes (based on Holmes et al., 2009; Lang, Moulds, & Holmes, 2009). Three scenes involved suicide and five scenes featured road traffic accidents. A further six scenes featured surgery, drowning, self-harm, an incident of bullying and genocide. The film was presented on a 17” VDU monitor in 32 bit colour. The viewing distance was approximately 0.5 m.

#### 3.1.4 Experimental tasks after the stressful film

Prior to the film all participants undertook training in both the visuospatial task and control-task as in Experiment 1. After the film, all participants undertook a structured 30-min break (as in Holmes et al., 2009; Holmes et al., 2010) and then completed a brief film reminder task in which they were presented with one neutral but recognizable image from each film clip for 3-s each in fixed order on the computer screen (Holmes et al., 2009). This procedure is in line with the broader literature on memory reconsolidation which suggests that retrieval of a memory triggered by a reminder cue may place that memory in a labile state, leaving that memory vulnerable to disruption (Hupbach, Gomez, Hardt, & Nadel, 2007; Schiller et al., 2010). Participants then completed their assigned experimental task condition for 10-min as in Experiment 1, with the exception that in the control-task participants counted backwards in sevens rather than threes to increase the difficulty of this task.

#### 3.1.5 Measures

The measures were identical to Experiment 1. Additionally, a recognition memory task (Holmes et al., 2009) was completed by participants to assess voluntary memory for the film at the follow-up session on Day 7. The task comprised 28 written statements regarding the film to which participants responded with either “true” or “false”. Fifteen statements were “true” and 13 were “false” and participants scored one point for each correct response. The maximum score was 28.

### 3.2 Results

The data were examined for potential univariate outliers. Two scores were more than 3 standard deviations from the mean and were changed to one unit larger (if the score was below the mean) or smaller (if the score was above the mean) than the next most extreme score in the distribution (Tabachnick & Fidell, 1996). An alpha level of 0.05 was used for all statistical tests.

#### 3.2.1 Group allocation, manipulation and compliance checks

There were no significant differences between the groups in age, BDI-II, STAI-T (*F*s < 1) or gender [*χ*^2^ (2, *N* = 75) = 0.11, *p* = .95] (Table 2). Ratings for attention paid to the film (*F* < 1) or personal relevance of the film [*F*(2, 72) = 1.98, *p* = .15] did not differ significantly between groups (Table 2). However, viewing the film resulted in the predicted increases in negative mood ratings across groups (Table 2). Repeated measures ANOVA on mood confirmed a significant main effect of time, *F*(1, 72) = 93.22, *MSE* = 158.72, *p* < .001, but no main effect of group, *F*(2, 72) = 0.93, *p* = .40, or time × group interaction, *F*(2, 72) = 0.48, *p* = .62.

In terms of task compliance, participants in the visuospatial condition tapped rapidly and accurately. The mean total number of key presses during the 10-min experimental phase was approximately 63 per min (*SD* = 25.2) and an accurate five-key sequence order was tapped on 81.9% of occasions (see Tables 2). For participants in the control-task condition the mean numbers counted was 179 (*SD* = 97.9) and the number of errors was 9.04 (*SD* = 7.8), meaning that correct responses were provided on 95% of occasions, again indicating good task compliance (Table 2).

There were no differences between groups in the accuracy reported by participants in maintaining the involuntary memory diary over the 7 days [*F*(2, 57) = 1.52, *p* = .23] (Table 2).

#### 3.2.2 Effects of experimental condition

**Fig. 2:**
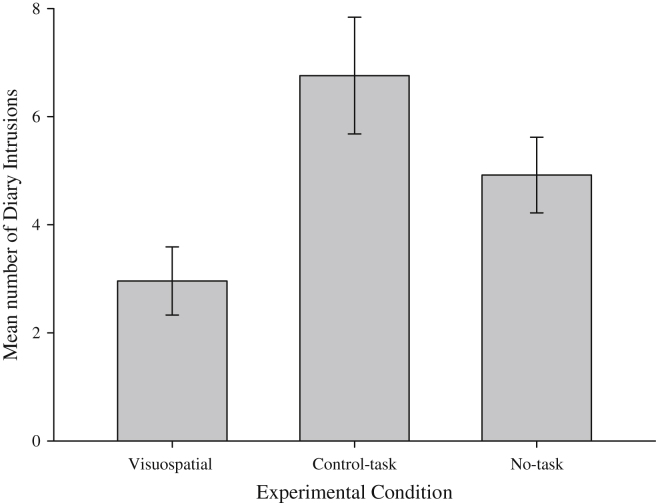
Experiment 2: Number of Involuntary Memories for each Experimental Condition. Error bars represent standard error of the mean.

One way ANOVA confirmed a significant difference in the number of involuntary memories of the stressful film between experimental conditions, *F*(2, 72) = 5.20, *MSE* = 90.3, *p* = .008, $\eta_{p}^{2}$ = 0.13, (Fig. 2). Independent samples *t*-tests identified significantly fewer involuntary memories in the visuospatial group compared to both no-task [*t*(48) = 2.06, *p* = .045, *d* = 0.6] and control-task [*t*(48) = 3.03, *p* = .004, *d* = 1.2]. The control and no-task conditions did not differ significantly [*t*(48) = 1.42, *p* = .16].

In contrast, there was no difference in recognition memory scores across groups [*F*(2, 72) = 0.41, *p* = .96] with very similar scores in each (overall mean score = 17.79; for group means see Table 2). Memory scores in each group were all significantly above chance; visuospatial [*t*(24) = 5.83, *p* = .001, *d* = 1.2], control-task [*t*(24) = 6.38, *p* = .001, *d* = 1.3] and no-task [*t*(24) = 6.79, *p* = .001, *d* = 1.2], indicating good but equivalent levels of performance.

## 4 General discussion

Both experiments confirmed that engaging in an established visuospatial task after viewing a stressful film (immediately post-film Experiment 1; 30-min post-film Experiment 2) led to fewer involuntary intrusive memories in the subsequent week compared to either a control-task (backwards counting) or a no-task condition. In comparison to the differential impact on involuntary memory, we found in Experiment 2 that voluntary memory for the film, assessed using a recognition task, did not appear to be affected. This is consistent with arguments that involuntary and voluntary memory are dissociable (Conway & Pleydell-Pearce, 2000). Our use of a verbal recognition task in the current studies was based on clinical models of involuntary memory development which suggest that there are two “streams” of processing (sensory-perceptual versus verbal) for stressful events (see Holmes & Bourne, 2008). However, as memory is a multifaceted cognitive domain, future research should explore the impact of visuospatial working memory tasks using a broader range of perceptual and conceptual memory tasks (e.g. Arntz et al., 2005; Halligan, Clark, & Ehlers, 2002). We believe it would be particularly important to assess voluntary memory for images from the film and compare this to the involuntary memories as recorded in the diary. In line with previous studies (Holmes et al., 2009; Holmes et al., 2010), we used a film reminder task prior to the experimental manipulation. Future studies should investigate whether this reminder task is critical to the efficacy of visuospatial tasks in modulating involuntary memory, as well as test whether this reminder of the stressful event may facilitate a time-window beyond the early consolidation period in which such cognitive tasks may be effective (Nader & Einarsson, 2010; Nader & Hardt, 2009; Schiller & Phelps, 2011).

We considered the potential explanations for our finding that only the visuospatial task reduced involuntary memories when completed post-film. It could be argued the visuospatial task was simply the most demanding task rather than specifically competing with sensory-perceptual processing. Direct comparison of the cognitive load of the visuospatial task compared to the control task is difficult in the context of the current study design and we cannot exclude the possibility of disparity in general task demands. However, even when the difficulty of the control-task was increased in Experiment 2 by requiring participants to count backwards in sevens rather than threes, there was still no evidence that it modulated involuntary memory. Future research should aim to establish the cognitive load of experimental and control tasks in order to allow direct comparison of task difficulty and the impact this may have on the modulation of involuntary memory. Findings were not attributable to differences in baseline characteristics (depression or anxiety scores), gender, mood effects, self-rated attention or personal relevance of the film, all of which were equivalent between groups.

Results progress our understanding of involuntary memory. First, they indicate that involuntary memories can be reduced by cognitive tasks performed after a stressful event, not only at encoding (Holmes et al., 2004). Our current methodology does not allow any firm conclusions to be drawn regarding the precise mechanisms involved (e.g. the role of retroactive interference, Dewar, Cowan, & Della Salla, 2007; Wixted, 2005) and this remains to be further explored. However, the possibility of modulating involuntary memory post-event is consistent with exciting work in cognitive neuroscience modulating fear memory during reconsolidation (e.g. Kindt, Soeter, & Vervliet, 2009; Schiller et al., 2010). Second, by comparing tasks known to tap into different cognitive domains, our results support the hypothesis that it is the imagery-based, sensory aspect of these memories that lead them to be intrusive, rather than the demand on general cognitive resources. If the visuospatial task was simply serving to increase cognitive load, we would expect recognition memory (as measured in Experiment 2) to be impaired (Craik, Govoni, Naveh-Benjamin, & Anderson, 1996), but this was not the case. We note however, recent findings indicating a relationship between involuntary memory and deliberate recall (Ferree & Cahill, 2009) and thus suggest that further research is required to explore in detail the effects of completing post-film cognitive tasks on a range of measures of voluntary memory. Finally, results aid the interpretation of our earlier finding that playing the computer game Tetris after a film reduces involuntary memories (Holmes et al., 2009; Holmes et al., 2010), and by using an established experimental task (rather than merely a computer game) provide convergent evidence that it may be the visuospatial nature of the task that is crucial.

It is not clear why verbal tasks sometimes increase, whilst others decrease or have no effect on intrusion development. Interestingly, previous studies have shown that some other types of experimental task (including such as counting backwards) may increase the development of involuntary memories when completed during the film (Bourne et al., 2010; Holmes et al., 2004). We did not find evidence in the current studies that counting backwards exerted such an effect. Indeed studies investigating negative autobiographical memories suggest working memory capacity rather than modality-specific properties of cognitive tasks may account for differences in results using verbal tasks (Engelhard et al., 2011; Gunter & Bodner, 2008; van den Hout et al., 2010; van den Hout et al., 2001). The pattern of results between the peri-film and post-film studies utilizing cognitive tasks suggest that the underlying mechanisms of encoding and consolidation of involuntary memories may differ. Our current data, and other that from other recent studies (Holmes et al., 2010) investigating post-event cognitive processing suggest this may be an important theoretical and clinical issue. However, theoretical frameworks from a cognitive behavioural therapy tradition (e.g., Brewin, Dalgleish, & Joseph, 1996; Ehlers & Clark, 2000) which focus on information processing during encoding of events have yet to elaborate upon the effects of post-event cognitive processing (Pearson, Ross, & Webster, 2012).

Once memories that tend to intrude involuntarily have been formed, there is evidence that their disruptive effects can be mitigated by effortful cognitive control, or ‘suppression’ (Anderson & Green, 2001; Anderson & Levy, 2009; Anderson et al., 2004; Depue, Curran, & Banich, 2007). Extensive work however has indicated that suppression may not be beneficial in healthy volunteers (Wegner & Gold, 1995; Wegner, Schneider, Carter, & White, 1987; Wegner, Shortt, Blake, & Page, 1990) and clinically, the approach has been argued to be contra-indicated (Holmes, Moulds, & Kavanagh, 2007; Holmes, Sandberg, & Iyadurai, 2010a, 2010b). The current experiments suggest that it may be possible to ameliorate unwanted involuntary memories of emotional events by performing relatively simple post-event cognitive tasks. The use of drugs such as propranolol after a negative event to disrupt memory consolidation has received considerable attention (Brunet et al., 2008; Kindt et al., 2009) and fear conditioning has been explored in this context (Schiller et al., 2010) Yet, little work to date has considered the possibility that even non-invasive cognitive tasks may reduce involuntary memories. We suggest that visuospatial tasks exert their influence via direct competition for the sensory-perceptual resources required for the successful consolidation of involuntary memories and thus, involuntary human memory may be manipulated by such tasks. Our findings have potential implications for preventing the development of involuntary autobiographical memories for events which we do not wish to come back to mind unbidden.

## Figures and Tables

**Table 1:** Experiment 1: Baseline, task manipulation and compliance measures from Experiment 1 for each experimental condition.

Variable	Visuospatial	Control-task	No-task
	*M* (SD)	*M* (SD)	*M* (SD)
Age	25.90 (5.64)	27.65 (9.98)	27.80 (9.45)
BDI-II	6.45 (7.79)	6.20 (6.34)	5.35 (5.13)
STAI-T	40.90 (12.53)	39.95 (8.66)	39.40 (9.54)
Pre-film mood	1.29 (1.20)	1.40 (1.51)	1.41 (1.20)
Post-film mood	2.25 (1.62)	2.84 (1.60)	2.52 (2.08)
Attention paid to the film	8.89 (1.01)	8.70 (1.17)	8.70 (1.27)
Personal relevance of film	4.07 (2.92)	3.49 (2.04)	3.50 (2.67)
Visuospatial tapping			
Total key presses	622.3 (138.34)		
No. of correct sequences	102.3 (22.81)		
Control-task			
Mean no. of responses		235.00 (63.67)	
Mean no. of errors		7.70 (9.61)	
Diary Accuracy	8.96 (1.09)	8.30 (1.28)	8.44 (1.20)

*Note:* BDI-II = Beck Depression Inventory; STAI-T = State Trait Anxiety Inventory – Trait.

**Table 2:** Experiment 2: Baseline, task manipulation, compliance measures and recognition memory scores from Experiment 2 for each experimental condition.

Variable	Visuospatial	Control-task	No-task
	*M* (SD)	*M* (SD)	*M* (SD)
Age	27.00 (11.20)	24.76 (8.38)	24.68 (7.22)
BDI-II	5.88 (5.89)	5.72 (6.42)	5.20 (4.69)
STAI-T	38.72 (9.03)	35.60 (11.31)	36.04 (7.37)
Pre-film mood	1.29 (1.44)	1.57 (1.44)	1.49 (1.33)
Post-film mood	3.05 (1.99)	3.76 (1.81)	3.71 (1.94)
Attention paid to the film	9.08 (0.86)	8.96 (1.21)	9.00 (0.87)
Personal relevance of film	4.24 (2.24)	4.52 (2.50)	5.48 (2.18)
Visuospatial tapping			
Total key presses	629.1 (252.25)		
No. of correct sequences	92.84 (42.97)		
Control-task			
Mean no. of responses		179.72 (97.89)	
Mean no. of errors		9.04 (7.59)	
Diary accuracy	8.16 (1.66)	7.56 (2.24)	8.40 (1.19)
Recognition memory	17.92 (2.89)	17.68 (3.16)	17.76 (2.95)

*Note:* BDI-II = Beck Depression Inventory; STAI-T = State Trait Anxiety Inventory – Trait.
